# SVMTriP: A Method to Predict Antigenic Epitopes Using Support Vector Machine to Integrate Tri-Peptide Similarity and Propensity

**DOI:** 10.1371/journal.pone.0045152

**Published:** 2012-09-12

**Authors:** Bo Yao, Lin Zhang, Shide Liang, Chi Zhang

**Affiliations:** 1 School of Biological Sciences, Center for Plant Science and Innovation, University of Nebraska, Lincoln, Nebraska, United States of America; 2 Department of Statistics, University of Nebraska, Lincoln, Nebraska, United States of America; 3 Systems Immunology Lab, Immunology Frontier Research Center, Osaka University, Suita, Osaka, Japan; University of Cincinnati College of Medicine, United States of America

## Abstract

Identifying protein surface regions preferentially recognizable by antibodies (antigenic epitopes) is at the heart of new immuno-diagnostic reagent discovery and vaccine design, and computational methods for antigenic epitope prediction provide crucial means to serve this purpose. Many linear B-cell epitope prediction methods were developed, such as BepiPred, ABCPred, AAP, BCPred, BayesB, BEOracle/BROracle, and BEST, towards this goal. However, effective immunological research demands more robust performance of the prediction method than what the current algorithms could provide. In this work, a new method to predict linear antigenic epitopes is developed; Support Vector Machine has been utilized by combining the Tri-peptide similarity and Propensity scores (SVMTriP). Applied to non-redundant B-cell linear epitopes extracted from IEDB, SVMTriP achieves a sensitivity of 80.1% and a precision of 55.2% with a five-fold cross-validation. The AUC value is 0.702. The combination of similarity and propensity of tri-peptide subsequences can improve the prediction performance for linear B-cell epitopes. Moreover, SVMTriP is capable of recognizing viral peptides from a human protein sequence background. A web server based on our method is constructed for public use. The server and all datasets used in the current study are available at http://sysbio.unl.edu/SVMTriP.

## Introduction

By secreting antibodies against antigens, B-cells play an important role in the immune system to fight an invasive pathogenic organism or substance. Antigenic epitopes are regions of the protein surface that are preferentially recognized by B-cell antibodies [1]. Prediction of antigenic epitopes is useful for the investigation on the mechanism of body's self-protection systems and could be helpful for the design of vaccine components and immuno-diagnostic reagents [2].

Usually, B-cell antigenic epitopes are classified as either continuous or discontinuous. A continuous (also called linear) epitope is a consecutive fragment from the protein sequence; a discontinuous epitope is composed of several fragments scattered along the protein sequence, but still form an antigen-binding interface in 3D. The boundary between continuous and discontinuous epitopes is vague; a continuous fragment in a discontinuous epitope can be considered as a continuous epitope. Currently, the majority of available epitope prediction methods focus on continuous epitopes due to the relative simplicity of the problem and the convenience of available investigation methods, in which the amino acid sequence of a protein is taken as the input. Such prediction methods are based upon the amino acid properties including hydrophilicity [3], [4], solvent accessibility [5], secondary structure [6], flexibility [7], and antigenicity [8]. In addition, based on the epitope databases such as IEDB [9], Bcipep [10], and FIMM [11], there are also some methods using machine learning approaches, such as Hidden Markov Model (HMM) [12], Artificial Neural Network (ANN) [13], and Support Vector Machine (SVM) [14], [15], to locate linear epitopes, such as PREDITOP [8], [16], PEOPLE [17], BEPITOPE [18], BepiPred [12], ABCPred [13], AAP [14], BCPred [15], BayesB [19], BEOracle/BROracle [20], and BEST [21].

In this work, a new linear B-cell epitope prediction method is developed using the SVM method to integrate the Tri-peptide similarity and Propensity scores (SVMTriP). SVMTriP is tested for varied epitope sequence lengths. With the five-fold cross-validation, SVMTriP achieves a sensitivity (Sn) of 80.1% and a precision (P) of 55.2% for sequences with 20 amino acids (AA), which are higher than those of AAP [14] and BCPred [15].

## Results

### Prediction performance

SVMTriP is trained and tested with different epitope lengths, and for each length, the SVM parameters have their independent optimal values. For example, for 20AA-length cases, SVMTriP reaches its optimal performance at *c* = 32, *g* = 0.05, and *p* = 0.5 for the SVM model with Sn  = 80.1% ±2.1% and P = 55.2% ±1.0% at the point with the maximal F-measure, 0.693. All results are shown in Table 1. Though, for different lengths of epitope sequences, SVMTriP has various points with the maximal F-measure, the precision values for different lengths are similar. The sensitivity increases significantly as the length of the epitope sequences becomes large. The range of the values of areas under the receiver operating characteristic curves (AUC) is from 0.674 to 0.702. Based on results of multiple evaluation methods (Table 1), SVMTriP for 18AA- and 20AA-length cases have the best performance. However, one may note a fact that most of experimental determined epitopes from IEDB [9] have less than 20 AA residues. A possible reason why SVMTriP favors long length of sequences is a long sequence may have more tri-peptides to show detectable frequency tendency. Another possibility is that the epitopic amino acid residues in experimentally determined epitopes are subsets of all real epitopic residues. Based on the testing results, 20AA is set as the default epitope length for SVMTriP to search for putative epitopes on the web server.

**Table 1:** Performance of SVMTriP models with different epitope lengths.

Length (AA)	Sn (%)	P (%)	F-measure	AUC
**10**	68.5±2.5	55.5±1.5	0.615±0.020	0.674
**12**	67.5±3.5	57.0±2.0	0.620±0.030	0.681
**14**	64.8±4.9	56.5±2.5	0.605±0.030	0.689
**16**	63.5±5.5	57.1±3.0	0.601±0.045	0.685
**18**	79.0±1.9	54.1±1.1	0.641±0.015	0.666
**20**	80.1±2.1	55.2±1.0	0.693±0.060	0.702

**Figure 1:**
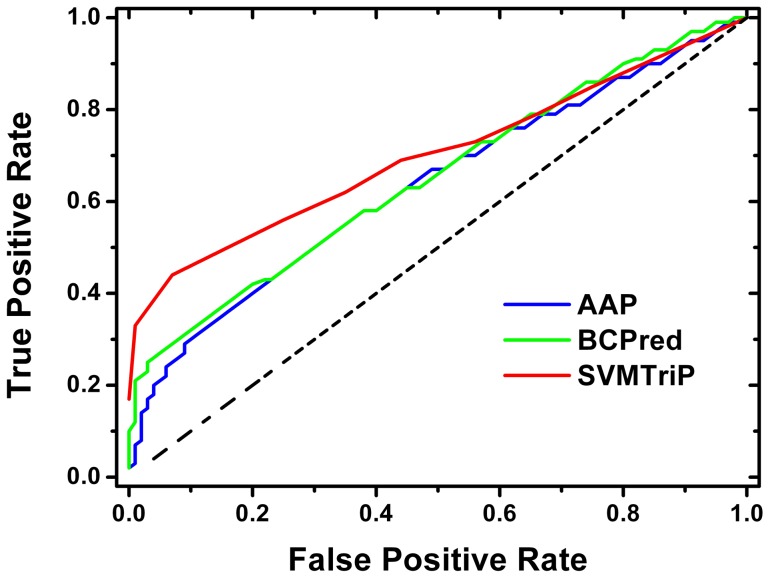
ROC curves for AAP, BCPred, and SVMTriP.

For comparison, AAP and BCPred are implemented locally based on their method descriptions [14], [15], trained/tested with the same dataset and the five-fold cross-validation procedure for 20AA case. The results are listed in Table 2. Compared with BCPred and AAP, SVMTriP has a similar precision value, but significantly improved sensitivity at the point with the maximal F-measure. Figure 1 shows the receiver operating characteristic curve (ROC) for three methods, from which one may notice that SVMTriP has significantly larger true positive rate than BCPred and AAP in the region of low false positive rate. The AUC values are 0.667, 0.667, and 0.702 for AAP, BCPred, and SVMTriP, respectively. The AUC value of SVMTriP is significantly higher than those from the other two methods; the p-values of comparison against AAP and BCPred are 2.17×10^−5^ and 1.58×10^−5^, respectively.

**Table 2:** Performance of different linear B-cell epitope prediction methods.

Methods	Sn (%)	P (%)	F-measure	AUC
**AAP** *	59.8±0.9	58.5±6.5	0.590±0.040	0.667
**BCPred** *	54.0±7.1	60.5±2.5	0.572±0.055	0.667
**SVMTriP**	80.1±2.1	55.2±1.0	0.693±0.060	0.702

* The results for AAP [14] and BCPred [15], are obtained by the software implemented locally.

### Top weighted tri-peptides

The prediction model relies on the occurring-frequency distribution of tri-peptides in the tri-peptide space, *i.e.* all combinations of any three amino acids. In Table 3, tri-peptides with top 20 weights in the optimal SVM model of 20AA-length epitopes are listed. All of the top ranked tri-peptides contain Glutamine or Proline, whereas the occurring frequencies of Glutamine and Proline in known linear epitopes (20AA) are only 8.1% and 6.84%, respectively. In the background of over all proteins, the occurring frequencies of Glutamine and Proline are 3.84% and 3.44% [22], which is not significantly different to the values in linear epitopes. However, the distribution patterns of the combined amino acids are quite different between epitopes and non-epitope peptides. Therefore, the tri-peptides containing Glutamine or Proline may play an important role in epitope recognition by B-cell antibodies. The algorithm of SVMTriP successfully utilized this difference to distinguish linear epitopes from other parts of protein peptides.

**Table 3:** Weights of tri-peptides in the optimal SVM model.

Tri-Peptide	Rank	Weight Score*	Tri-Peptide	Rank	Weight Score*
QQP	1	503251.79	GQQ	11	121677.62
PQQ	2	488627.71	QPY	12	116598.60
QPQ	3	367386.40	YPQ	13	113237.37
QPF	4	246462.39	QQF	14	81709.59
FPQ	5	234868.65	PYP	15	79191.37
PQP	6	231353.73	FQQ	16	77357.97
QGQ	7	153161.76	PPP	17	76320.05
PFP	8	151840.02	QPP	18	64756.05
QQQ	9	128930.20	QFP	19	63814.16
QQG	10	122291.90	PPQ	20	63173.33

* Weight scores are calculated by the formula w = ∑ α_ i_ *x_i_*. Here α is dual representation of the decision boundary; and *x_i_* (i = 0, 1, 2…n) is vector described in SVM model. Both α_ i_ and *x_i_* are available in model file.

### Tendency of prediction between virus and human proteins

**Figure 2:**
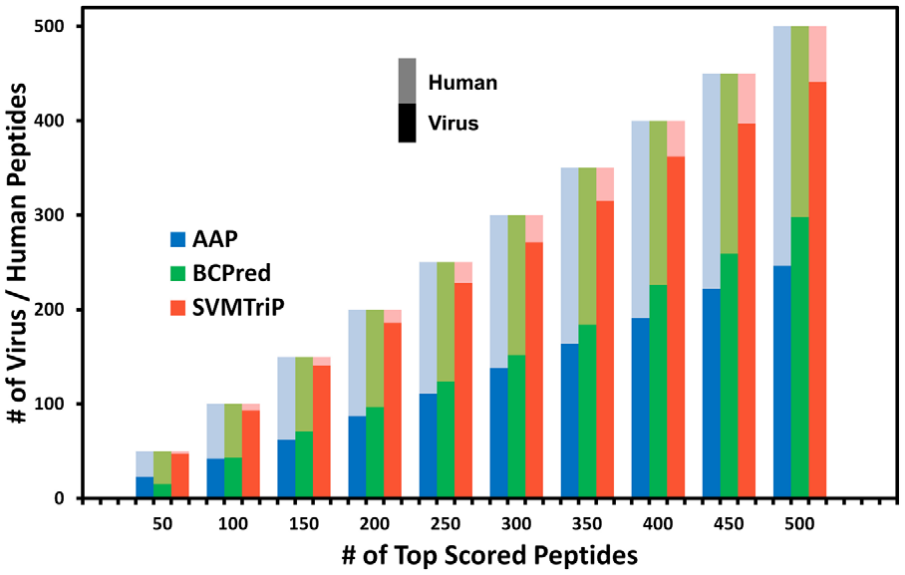
Tendency test for BCPred, AAP, and SVMTriP. Three bars at the same point on the x-axis are the results for APP (blue), BCPred (green), and SVMTriP (red), respectively. In the same bar, the light part is for the number of returned human peptide, and the dark part is for virus. For example, at the point of 400 returned peptides, the dark part in the red bar is 362, which means that 362 viral peptides are return in all 400 peptides by SVMTriP, and the light red part represents 38 human peptides.

Independent test of different epitope prediction methods is challenging because of the limited number of known epitopes. In this study, we devise an alternative independent test method. In the training set, most epitopes are from virus or bacteria, and their corresponding antibodies are mainly human antibodies. A basic property of the human immune system is the capability to distinguish any pathogenic agents, viral or bacterial, from the innate structures of the human being. All known B-cell epitopes in the training set came from the response of whole immune system, including the response of CD4 T helper cells. In order to simulate the human immune system, a successfully trained epitope prediction method should act the same, *i.e*. be able to distinguish pathogenic proteins from human proteins. In other words, the virus proteins should be preferentially more highly scored than human proteins by a successful prediction algorithm. To implement this test, 10^5^ 20AA-length peptides are collected from virus and human proteins: 5×10^4^ peptides are randomly selected from 391,466 virus proteins and others from 81,967 human proteins in the Refseq protein database [23]. AAP, BCPred, and SVMTriP are applied to these virus and human peptides, and top-ranked peptides are returned. The fractions of virus peptides in different numbers of returned peptides are shown in Figure 2. In most cases, the three methods returned more virus peptide than human peptides within the top-ranked peptides. SVMTriP, however, selected higher percentage of virus peptides than both AAP and BCPred. For example, in total 400 top-ranked peptides returned by SVMTriP, 90.5% of them, *i.e.* 362, are virus peptides. There are 47.8% (191), 56.5% (226) virus peptides returning by AAP and BCPred, respectively. This indicates the exceptional ability of SVMTriP to distinguish epitopic and non-epitopic peptides.

## Discussion

### Prediction with tri-peptide propensity alone

The propensity of tri-peptide alone is tested and the result is shown in Table 4. The prediction sensitivity and precision are 56.5% bad 61.0%, respectively, similar to those of AAP, which is based on bi-peptide propensity and yielded a sensitivity of 59.8% and precision of 58.5% for the same test set. This result indicates that combining similarity scores is essential for the tri-peptide model to achieve a better performance.

**Table 4:** Comparison among the tri-peptide subsequence models with or without propensity.

Kernels			Sn (%)	P (%)	F-measure
Tri-peptide	Propensity only	N.A.	56.5±12.5	61.0±6.3	0.584±0.085
Tri-peptide	w./o. Propensity	Blosum62	54.5±6.5	60.5±1.5	0.573±0.035
		PAM160	55.0±7.2	61.1±1.8	0.578±0.040
	w./Propensity	**Blosum** **62** *	**80.1±** **2.1**	**55.2±** **1.0**	**0.693±** **0.060**
		PAM160	69.3±10.0	58.5±3.5	0.633±0.050
AA_A pattern	w./o. Propensity	Blosum62	54.8±6.8	60.5±1.5	0.579±0.040
		PAM160	55.2±7.1	61.3±2.0	0.577±0.045
	w./Propensity	Blosum62	60.5±5.5	57.5±2.5	0.589±0.040
		PAM160	59.5±5.5	57.5±1.5	0.585±0.035
A_AA pattern	w./o. Propensity	Blosum62	55.5±8.5	60.6±2.2	0.581±0.050
		PAM160	55.2±8.1	60.5±1.5	0.577±0.055
	w./Propensity	Blosum62	60.5±6.5	57.5±1.5	0.590±0.040
		PAM160	59.5±5.5	57.5±1.5	0.585±0.025

* The corresponding model is defined as SVMTriP.

### Prediction with tri-peptide similarity alone

The tri-peptide similarity scores can be calculated with either Blosum62 or PAM160 matrixes. The performance of two different matrices for the tri-peptide model is evaluated with the same procedure of the five-fold cross-validation for 20AA-length epitopes. The results are shown in Table 4. Without the propensity score, using Blosum62 matrix shows similar performance as using the PAM160. However, when combined with the propensity score, Blosum62 matrix leads to a higher prediction performance.

### Discrete tri-peptide subsequence models

We also implement a method that uses the space of tetra-peptide subsequence with one mismatch, *i.e.* discrete tri-peptide subsequences. For this case, the subsequences are considered in patterns like A_AA or AA_A, where ‘A’ represents the amino acid residue to be considered, and ‘_’ represents the residue position that will be ignored in the comparison. The number of SVM attributes is still 20^3^, which is identical to that of the tri-peptide model. Interestingly, without considering propensity scores, the subsequence models of A_AA and AA_A patterns have similar sensitivity and precision with the tri-peptide model. However, the combination of similarity and propensity of the tri-peptide model significantly enhances the performance, while addition of the propensity does not increase sensitivity or precision for A_AA and AA_A patterns. The result is shown in Table 4. This finding indicates that the propensity is more important for the tri-peptide model than the discrete tri-peptide subsequence model.

### Conclusion

The performance for linear B-cell epitope prediction is improved by concurrently using similarity and propensity of the tri-peptide model. Combination of similarity and propensity gives rise to an excellent performance for the tri-peptide model, but does not for the discrete tri-peptide subsequence model. SVMTriP finally achieved the AUC value of 0.702 and, at the point with the maximal F-measure, Sn  = 80.1% and P = 55.2%. Further more, SVMTriP is capable of distinguishing virus peptides from human ones, and hence, has a higher chance to correctly predict linear B-cell epitopes. The web server, trained models, and all datasets are available at http://sysbio.unl.edu/SVMTriP.

## Materials and Methods

### Datasets

The dataset is constructed by extracting non-redundant linear B-cell epitopes from IEDB [9], because it is frequently updated and has a large number of linear epitopes. Total of 65,456 B-cell linear epitopes are downloaded from IEDB (version June 11th, 2012). The identical epitopes and those possibly related to T-cell are removed. The full-length sequences of corresponding epitopes are also collected. The various lengths of epitope sequences, including 10AA, 12AA, 14AA, 16AA, 18AA, and 20AA, are extracted by trimming the long experimental measured epitopes or attaching more amino acid residues to both ends of short epitopes according to the full-length sequences. For a given length, epitope sequences with ≥30% similarity, measured by BLAST [24], are clustered together and only one of them is kept as an epitope sequence in the dataset. Finally, the dataset for each length has a total of 4925 non-redundant epitope sequences. For the negative dataset, the same numbers of equal-length sub-sequences are extracted from the non-epitopic segments in the corresponding antigen sequences.

### Support Vector Machine Setup

#### Attribute encoding

The tri-peptide subsequence space is used to encode the SVM attributes. This kernel has a space of 20^3^ attributes for both tri-peptide substring and propensity. The score of *i*-th attribute, *K* **^(^** ^***i*)**^, is defined as the tri-peptide subsequence similarity kernel modulated by its corresponding tri-peptide propensity. Please see Equation (1):
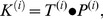
(1)


where *K* **^(^** ^***i*)**^ denotes the score of the *i*-th attribute, *T* ^(*i*)^ denotes the *i*-th tri-peptide subsequence similarity kernel, and *P* ^(*i*)^ denotes corresponding tri-peptide subsequence propensity of *i*-th tri-peptide subsequence. The tri-peptide subsequence similarity kernel is defined as:
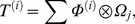
(2)


where Φ^(i)^ denotes the tri-peptide that represents the *i*-th attribute, Ω*_j_* denotes the *j*-th tri-peptide in the tri-peptide subsequence space for the input sequence. The symbol “∶” denotes getting the similarity score of any two corresponding tri-peptide, *i.e.* sum of three similarity scores for three amino acid pairs from a Blosum/PAM matrix. For example, assuming the length of a given epitope candidate is 20 AA, the tri-peptide subsequence similarity kernel for the *i*-th attribute is generated by summing over similarity scores of the 18 pairs of tri-peptides; each pair consists of one tri-peptide from the input sequence and the tri-peptide represents *i*-th attribute from the tri-peptide subsequence space. This subsequence kernel was previously used to predict protein subcellular localization by Lei and Dai [25]. The propensity of tri-peptide subsequence representing the *i*-th attribute is calculated as in Equation (3):
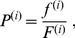
(3)where *f(i)* is the frequency of i-th type of tri-peptide in the positive epitopes, and *F(i)* is the frequency of i-th type of tri-peptide in 5×104 protein sequences randomly selected from the Refseq database [23].

#### Training/Prediction procedure

The SVM training in this work uses an SVM package, SVM^light^, implemented by Joachims (http://svmlight.joachims.org/) [26]. All SVM parameters are optimized by a grid search (*c* = 2^−10∼−1^, *g* = 2^−12∼−3^, and *p* = 2^−5∼−2^). For each grid point of the triplets, a five-fold cross-validation procedure is employed to evaluate the performance of the trained SVM model. To carry out the five-fold validation procedure, the total of 4925 positive epitopes are split into five groups, and any two-epitope sequences from two different groups do not have sequence similarity more than 20%. At each triplet point, the maximum F-measure is calculated. The optimal parameter set has the largest value in all points by the maximum F-measures. During the procedure of five-fold validation, five test results are used to calculate the mean values and 95% confidence intervals of sensitivity, precision, and the maximal F-measure.

For the application on the online server, the prediction model is obtained by training the whole dataset with the same numbers of positive and negative epitopes. To predict a given full-length protein sequence, the sliding window method is employed to obtain subsequences with variable lengths, including 10AA, 12AA, 14AA, 16AA, 18AA, and 20AA. For each subsequence, SVMTriP calculates its score, and a positive score indicates that the subsequence is a putative antigenic epitope.

### Evaluation methods

The statistical terms, sensitivity (Sn), precision (P), and F-measure, are defined in the following equations:
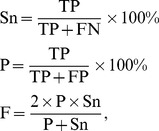
where TP, TN, FP, and FP stand for true positive, true negative, false positive, and false negative, respectively. F-measure is used to determine the optimal prediction results. A java program available at http://pages.cs.wisc.edu/~richm/programs/AUC/ is used to calculate the AUC. The online tool StAR [27], [28] is used to test whether the difference between ROC curves resulting from two methods is statistically significant.
